# Effects of probiotics on non-alcoholic fatty liver disease: a review of human clinical trials

**DOI:** 10.3389/fnut.2023.1155306

**Published:** 2023-06-30

**Authors:** Chujin Cao, Mengxia Shi, Xiuru Wang, Ying Yao, Rui Zeng

**Affiliations:** ^1^Division of Nephrology, Tongji Hospital, Tongji Medical College, Huazhong University of Science and Technology, Wuhan, China; ^2^Division of Nutrition, Tongji Hospital, Tongji Medical College, Huazhong University of Science and Technology, Wuhan, China; ^3^Key Laboratory of Organ Transplantation, Ministry of Education, Chinese Academy of Medical Sciences, Wuhan, China; ^4^NHC Key Laboratory of Organ Transplantation, Chinese Academy of Medical Sciences, Wuhan, China

**Keywords:** non-alcoholic fatty liver disease, gut microbiota, gut-liver axis, probiotics, randomized clinical trial

## Abstract

Non-alcoholic fatty liver disease (NAFLD) is a global public health issue, of which the prevalence is about 25% worldwide. The incidence of NAFLD is increasing in patients with obesity, type 2 diabetes (T2DM) and the metabolic syndrome. The crosstalk between gut microbiota and metabolism-related diseases has been raised great concern. Patients with NAPLD were observed with disruption of gut microbiota. Several researches showed that gut microbiota was the determination in the progression of NAFLD by the experiments using fecal microbiota transplants. The application of probiotics, as one of the most important strategies for the regulation of gut microbiota disorder, have been explored whether it is beneficial to gut-related diseases of intestine-distal organs. Some probiotics were showed to improve the liver parameters and phenotype in patients with NAFLD. The oral intake of them might become the effective management for the prevention and treatment of NAFLD. In this review, we summarized the human clinical trials focusing on the effects of probiotics on NAFLD to give some evidential reference for the administration of NAFLD.

## 1. Introduction

Non-alcoholic fatty liver disease (NAFLD) is a global public health care with a prevalence of 25.2% worldwide, 27.4% in Asia (1, 2) and over 33% in China (3, 4). The main characteristics of NAFLD is the accumulation of lipids in hepatocytes over 5% of liver weight without excessive alcohol intake (5). The progression of NAFLD encompasses three stages of different but correlative pathogenic conditions. The first stage is simple steatosis characterized by the onset of liver fat accumulation in hepatocytes. The second stage is non-alcoholic steatohepatitis (NASH), which is the outcome of hepatocyte injury in the process of inflammation, ballooning degeneration and slight collagen deposition (6, 7). Without prevention of NASH, the progressive form may gradually move into the third stage, in which it results in cirrhosis and hepatocellular carcinoma, eventually leading to liver failure (8). The epidemiologic researches have shown that approximately 10–20% of NAFLD population makes a progression to NASH (9). Therefore, the prevention of NAFLD progression in the early stage is crucial for its clinical therapy. Despite the abundance of clinical trials on the pharmacological treatments for NAFLD, no specific drugs are available for NAFLD, and in the late phase of cirrhosis, surgical procedures seem pointless except for hepatic transplantation (10). NAFLD is a multifactorial disease, of which the pathogenic mechanisms are still under a limited understanding and the accurate non-invasive biomarkers remain to be lacking (11). Therefore, there is no specific pharmacological therapy available to treat NAFLD.

In general, NAFLD is a metabolic dysbiosis and has been renamed metabolic-associated fatty liver disease (MAFLD), recently (11). Here, we still use “NAFLD” in this review to avoid confusion. NAFLD is associated with lipid and glucose metabolism, and patients with obesity and type 2 diabetes mellitus (T2DM) have the increased risk of NAFLD (12). Thus, insulin resistance is reported to be the main mechanism of NAFLD development (13). In the current perception, “multiple-hit theory” is the consensus on the pathogenesis of NAFLD (14, 15). Several researches have been completed to explore the pathogenic mechanisms of NAFLD, including endothelial dysfunction (16), excessive hepatic lipid-induced inflammatory responses (17–19) and generation of reactive oxygen species (ROS) (20–22). All those result in lipid overload in liver. The accumulation of fat is due to the imbalance of fatty acid (FA) delivery to the liver, lipid synthesis and consumption, and triglyceride (TG) export out of the liver (23). The excess dietary FA, *de novo*-lipogenesis and FA uptake from circulation contribute to the increased FA import. The synthesis of TG, lipoprotein secretion, lipid droplets formation and lipophagy are the determination for TG export out of the liver (24). In addition, because of the close relationship between lipid metabolism and energy production, the role of mitochondria in NAFLD progression has been received attention, including the regulation of production of FA oxidation and ROS, the activation of the inflammasome and cell apoptosis, and interaction of mitochondria with other cell organelles (25, 26). In addition, patients with NAFLD are more likely to suffer extra hepatic cancer, such as the elderly bladder cancer (13). Currently, lifestyle interventions, diet, and exercise are the recommended but only subsidiary interventions for NAFLD treatment (27). Recently, natural products, such as spirulina, oleuropein, garlic and so on, have become an alternative approach in the treatment of NAFLD due to their large availability, low-cost and safety. However, the efficacy in patients is not clear with the existed animal experiments only (28). Several pharmacological treatments with anti-diabetic and anti-lipidemic effects have been confirmed to have drawbacks, such as Vitamin E and pioglitazone (29). The drugs, which are currently in clinical trials but only for the treatment of noncirrhotic NASH, include the farnesoid X receptor agonist obeticholic acid (OCA), the thyroid hormone receptor THRβ agonist (Resmetirom), and Aramchol (bile acid and FA conjugate, cholic acid–arachidic acid) (30–32). Therefore, to explore the new therapeutic strategies is crucial to effective treatments for NAFLD.

Recently, based on the theory of “gut-liver” axis, gut microbiota and gut-derived metabolites have been proved in the occurrence and progression of NAFLD. The improper living lifestyle, especially diets, NAFLD itself and the complications all contribute to intestinal dysbiosis, including the disorder of gut microbiota structures, gut-derived metabolite dysbiosis and the disruption of intestinal barrier. These together result in exacerbating NAFLD progression (33–36). Therefore, redressing intestinal imbalance may be a potential therapeutic strategy for NAFLD treatment.

Probiotics, one of the intestinal flora regulating drugs, are non-pathogenic live microorganisms for gut health and generally applicated in diarrhea (37, 38) and malnutrition (39, 40). In recent, supplements of probiotics have been reported to play a beneficial role in more intro-intestinal or extro-intestinal diseases, such as inflammatory bowel disease (IBD) (41, 42), rheumatic arthritis (43, 44), T2DM (45, 46), chronic kidney disease (CKD) (47, 48), NAFLD (49, 50) and so on. Probiotics reform gut microbiota imbalance (51), improve lipid and glucose profiles (52, 53), reserve the intestinal barrier integrity (54), reduce inflammation (55) and inhibit oxidative stress (56), by which NAFLD theoretically may be prevented effectively. The exact effect of probiotics on NAFLD in clinical practice is still lacking, although abundant animal experiments have been completed to explore the mechanisms of probiotics for NAFLD treatment.

In this review, firstly, we described the manifestation of gut dysbiosis in patients with NAFLD. Next, we summarized the clinical trials of probiotics on NAFLD to evaluate the practical effects and discuss the therapeutic administrations. Finally, we analyzed the challenges of the applications of probiotics for NAFLD treatment and the prospect in this promising field.

## 2. The role of “gut-liver axis” on NAFLD

**Figure 1:**
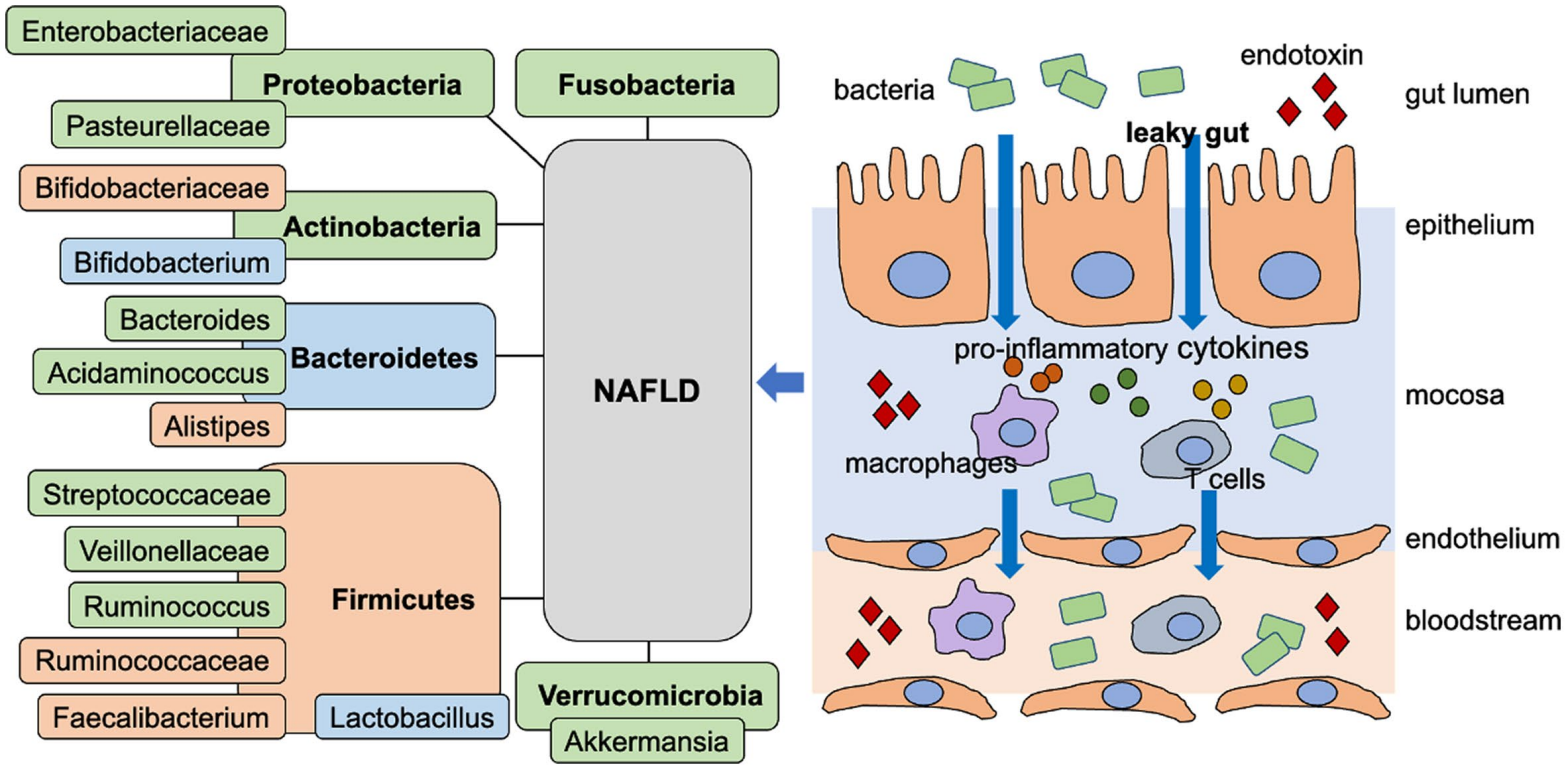
The role of “gut-liver axis” on NAFLD. Several clinical trials revealed that Fusobacteria, Verrucomicrobia, Proteobacteria, Actinobacteria increased, but Firmicutes decreased at phylum level. However, the trend of Bacteroidetes was in conflict. Streptococcaceae, Veillonellaceae, Bacteroides, Pasteurellaceae and Enterobacteriaceae increased, but Bifidobacteriaceae and Ruminococcaceae decreased at family level. Ruminococcus, Acidaminococcus and Akkermansia increased, but Faecalibacterium and Alistipes decreased at genus level. However, the changes of Bifidobacterium and Lactobacillus were in conflict. Gut dysbiosis in NAFLD led to the leaky gut with increased gut permeability, which enabled bacteria and endotoxins into gut mucosa, and then into blood circulation. The endotoxins and bacteria activated immune responses and induced the low-grade inflammation. The green frame represents the increased change, the orange frame represents the decreased change, and the blue frame represents the change in conflict.

It is confirmed that patients with NAFLD suffer from gut dysbiosis, which is characterized by alterations of gut microbiota compositions and gut-derived metabolites, dysfunction of gut barriers, and microbe translocation (33, 34, 57). Several observational researches have described the composition of gut microbiota in patients with NAFLD. However, the results are not consistent on account of the differences in study design, object region and sequencing method. Most importantly, the pathological progression of NAFLD may contribute to the changes of gut microbiota structure (34). In this part, we summarized the manifestations of gut dysbiosis in NAFLD and the mechanisms based on “gut-liver axis” for NAFLD pathogenesis, which were showed in Figure 1.

### 2.1. Gut dysbiosis in patients with NAFLD

Under the process in NAFLD without fibrosis progression, Verrucomicrobia, Fusobacteria, and Proteobacteria were increased compared to the healthy group (58, 59). Hoyles et al. found that Actinobacteria was significantly correlated, whereas Firmicutes was significantly anti-correlated with NAFLD by molecular phenomics and metagenomics (58). Another observational study showed that non-obese patients with NAFLD had 20% more Bacteroidetes and 24% less Firmicutes compared to healthy control (60). However, Chierico et al. discovered that Bacteroidetes was reduced in NAFLD (61). The discordant results may be explained by the differences of dietary style and sample alterations.

On the status of liver fibrosis, the changes of gut microbiota composition remain unclear, and several studies have conclusions in conflict. Loomba et al. revealed that Proteobacteria was increased, whereas Firmicutes was decreased in progression from NAFLD to advanced fibrosis (62). Another study supported above conclusion and showed that the proportion of Bacteroidetes was significantly reduced, whereas Proteobacteria and Fusobacteria were highly abundant in the fibrosis group (63). In addition, at family level, the consensus was that Streptococcaceae, Enterobacteriaceae, Pasteurellaceae, and Veillonellaceae were increased not only in NAFLD but also in advanced fibrosis group (59, 63–65). Ruminococcaceae and Bifidobacteriaceae were reduced in NAFLD group (65). The changes at genus levels were more bewildering. It was confirmed that Bacteroides, Ruminococcus, Acidaminococcus, and Akkermansia were increased, whereas Alistipes and Faecalibacterium were reduced in NAFLD (64–66). Besides that, some bacterial genus exhibited an ambiguous alteration, such as Bifidobacterium and Lactobacillus. The researches associated with Clostridium and Streptococcus are lacking (64, 65). Therefore, the determination of gut microbe composition in patients with NAFLD remains to be difficult for consistence due to the influence of many factors, including ages and lifestyle of subjects, severity of NAFLD and accompany of complications.

Although patients with NAFLD have the complicated gut microbe alterations, the researches on the metabonomic revealed the consistent results that the altered microbiota may be the important factor of contributing to NAFLD by the mechanism of impaired production of short-chain fatty acids (SCFAs) (67). Rau et al. found that NAFLD patients had higher fecal SCFA levels, and the main differential bacteria was SCFA-producing bacteria. Higher fecal propionate and acetate levels were related to lower resting regulatory T-cells (rTregs) and higher Th17/rTreg ratio in peripheral blood, which showed the mechanism by the interaction of metabolites and immunoregulation (67). In consideration of the accompany of gut microbiota changes, regulation of these bacteria and microenvironment may be the novel strategy for NAFLD therapy.

### 2.2. The mechanism based on “gut-liver axis” for pathogenesis of NAFLD

The “gut-liver axis” emphasizes the bidirectional relationship between gut and liver, which is established by the special anatomical interactions. Portal vein enables the delivery of gut-derived products to liver, and the feedback route of bile and antibody secretion is from liver to gut. Generally, gut dysbiosis contributes to the pathogenesis of NAFLD by leading to leaky gut, bacterial translocation and low-grade inflammation related to endotoxemia (68, 69).

The gut barrier is the functional and anatomical structure as a playground for “gut-liver axis,” which limits the systemic dissemination of microbes and toxins into circulation and liver (68). Patients with NAFLD were found to accompany with the disruption of gut barrier manifested as the increased intestinal permeability, which may partly result from the high-fat diet (69). We describe the gut barrier with increased permeability as leaky gut. The leaky condition serves as the prerequisite of bacterial translocation and endotoxin leakage (70). The endotoxin links gut barrier impairment with systemic low-grade inflammation by promoting immune activation. For example, increased endotoxins enable macrophages to secret pro-inflammatory cytokines partly by toll-like receptor (TLR) signaling pathways (71). Gut-derived Th17 contributes to the migration of neutrophils, the maintenance of gut homeostasis and the limitation of lipopolysaccharide (LPS) translocation to visceral adipose tissue by interleukin-17 (IL-17)/interleukin-17 receptor (IL-17R) axis (72, 73). In addition, bacteria or its product translocation into the blood circulation and liver is another result due to leaky gut, which is presented as antigens contributing to low-grade inflammation and regulated by β-catenin activation in endothelial cells (69). Based on the important role of “gut-liver axis” on NAFLD progression, restoring gut dysbiosis have become the promising treatments in clinical practice.

## 3. Effects of probiotics on NAFLD in clinical trials

Probiotics supplement is considered as one of the effective interventions to regulate gut microbiota. Several animal experiments have demonstrated probiotics were beneficial to NAFLD by reducing inflammation, the hepatic triglyceride, total body, and visceral adipose tissue weight, and by improving the insulin resistance (74, 75). Nevertheless, the clinical trials on effects of probiotics on NAFLD remain to be lacking. Here, we summarized recent clinical results showed in Table 1 to evaluate the exact effects of probiotics laying the foundation for further studies.

**Table 1:** The summary of clinical trials on effects of probiotics for NAFLD patients.

Research type	Research objects	Sample size	Probiotics	Duration	Outcomes related to gut dysbiosis	Outcomes related to NAFLD progression	References
RCT	obese children with biopsy-proven NAFLD	44	VSL#3	4 months	not mentioned	The decreased index: percentage of moderate and severe FL, BMI. The increased index: percentage of none and light FL, GLP and aGLP1. No changes with significant differences: TG, HOMA and ALT.	(76)
	patients with NAFLD	140	unclear probiotics	3 months	Conditions of fecal flora in the probiotic group were better than those in the placebo group	The decreased index: ALT, AST, GGT, TC, TG, HOMA-IR, NAS.	(77)
	patients with NAFLD	110	unclear probiotics	12 weeks		The decrease index: triglyceride, ALT, AST, GGT, AKP and hs-CRP.	(78)
	obese children with sonographic NAFLD	64	probiotic capsule (containing *Lactobacillus acidophilus* ATCC B3208, *Bifidobacterium lactis* DSMZ 32269, *Bifidobacterium bifidum* ATCC SD6576, *Lactobacillus rhamnosus* DSMZ 21690)	12 weeks	not mentioned	The decrease index: ALT, AST, cholesterol, LDL-C, TG, and waist circumference. The increased index: the percentage of normal liver sonography. No change with significant differences: weight, BMI, and BMI z score.	(79)
	patients with NAFLD	200	the live “combined Bifidobacterium Lactobacillus and Enterococcus powder,” two live “combined *Bacillus subtilis* and Enterococcus” or both probiotics	1 month	Compared with before treatment, fecal flora in combined groups was all reduced, but it was comparable before and after treatment in control group.	The decreased index: blood lipids and glucose, ALT, AST, TNF-α. The increased index: HMW-APN. No change with significant differences: fatty liver by ultrasound.	(80)
	patients with histology-proven NASH	20	probiotic formula containing *Lactobacillus plantarum*, Lactobacillus deslbrueckii, *Lactobacillus acidophilus*, *Lactobacillus rhamnosus* and *Bifidobacterium bifidum*	6 months	not mentioned	The decreased index: IHTG, AST. No change with significant differences: BMI, waist circumference, glucose and lipid levels	(81)
	patients with NASH fed a low-fat/low-calorie diet	75	the probiotic cocktail once daily	12 weeks	The composition of stool microbiota in probiotic-treated patients demonstrated a shift toward a normal pattern for all bacterial species examined.	The decreased index: serum ALT, liver stiffness, BMI, serum cholesterol. No changes with significant differences: GGT	(82)
	patients with NAFLD (diagnosed by liver biopsy)	28	one tablet per day with *Lactobacillus bulgaricus* and *Streptococcus thermophilus*	3 months	not mentioned	The decreased index: ALT, AST and γ-GT. No changes with significant differences: anthropometric parameters and cardiovascular risk factors.	(83)
	patients with NAFLD	35	2 sachets VSL#3^®^ probiotic or placebo	twice daily for 10 weeks	not mentioned	No change with significant difference: biomarkers of cardiovascular risk and liver injury	(84)
	T2DM patients with NAFLD	58	live multi-strain probiotic “Symbiter”(concentrated biomass of 14 probiotic bacteria genera Lactobacillus: Lactococcus, Bifidobacterium, Propionibacteriu, Acetobacter)	8 weeks	not mentioned	The decreased index: FLI, LS, AST, GGT, TNF-α and IL-6. No change with significant differences: serum lipids, IL-1β, IL-8, and IFN-γ.	(85)
	patients with NAFLD	42	2 capsules per day of probiotics	8 weeks	not mentioned	The decreased index: insulin, insulin resistance, TNF-α, and IL-6. No changes with significant differences: TNF-α	(86)
	obese NAFLD patients	68	probiotic mixture included 6 bacterial species	12 weeks	not mentioned	The decreased index: body weight, total body fat, IHF fraction, TG.	(87)
	adult NASH	46	sachets of probiotic mix (*Lactobacillus acidophilus*, *Lactobacillus rhamnosus*, *Lactobacillus paracasei* and *Bifidobacterium lactis*)	24 weeks	not mentioned	No change with significant differences: BMI, lipid and glucose profile, atherogenic indexes including PAI-1 and miR-122 levels.	(88)
	patients with NAFLD	39	MCP^®^ BCMC^®^ Strains	6 months	stabilize the mucosal immune function and protect NAFLD patients against increased intestinal permeability	No change with significant differences: hepatic steatosis and fibrosis levels as measured by transient elastography, LiverFAST analysis (steatosis, fibrosis and inflammation scores), ALT, TC, TG, fasting glucose, CD4+ T lymphocytes, CD8+ T lymphocytes and ZO-1.	(89)

RCT, randomized, controlled trial; NAFLD, non-alcoholic fatty liver disease; NASH, non-alcoholic steatohepatitis; T2DM, type 2 diabetes; FL, fatty liver; BMI, body mass index; GLP, glucagon-like peptide; TG, triglyceride; HOMA, homeostasis model assessment; ALT, alanine aminotransferase; AST, aspartate aminotransferase; GGT, glutamine transferase; TC, total cholesterol; HOMA-IR, homeostasis model assessment of insulin resistance; NAS, NAFLD activity score; AKP, alkaline phosphatase; hs-CRP, high-sensitive C-reactive protein; LDL-C, low-density lipoprotein cholesterol; TNF, tumor necrosis factor; HMW-APN, high molecular weight adiponectin; IHTG, intrahepatic triglyceride, content; GT, glutamine transaminase; FLI, fatty liver index; LS, liver stiffness; IL, interleukin; IFN, interferon; IHF, intrahepatic fat; PAI, plasminogen activator inhibitor; ZO-1, Zonula Occludens-1.

Randomized, double-blinded and placebo-controlled trials were commonly undertaken. In the aspect of NAFLD severity, the probiotics decreased the percentage of moderate and severe FL but had no effect on reducing the levels of ALT, AST, TG and insulin resistance (76–83). However, another study showed that VSL#3^®^ probiotic supplementation did not significantly improve liver injury as well as the cardiovascular risk (84). For liver injury, a 3-month clinical research provided the positive evidence that ALT, AST, GGT, TC, TG, HOMA-IR, NAS, and conditions of fecal flora in the probiotic group were better than those in the placebo group, and the probiotic group was better after treatment than before, which showed that probiotics can partly improve liver functions, glucose and lipids metabolism, hepatic fatty deposition in patients with NAFLD (77). Another 12-week study showed that probiotic supplementation was able to decrease TC, ALT, AST, GGT, and AKP compared to control group. Hs-CRP significantly decreased after intervention, however, there was no significant difference compared to control group (78). The multi-probiotic “Symbiter” concentrated biomass of 14 probiotic bacteria genera Bifidobacterium, Lactobacillus, Lactococcus and Propionibacterium. T2DM patients with NAFLD were administrated for 8 weeks. For liver injury, the muti-probiotics reduced fatty liver index (FLI) and the level of serum alanine aminotransferase (AST) and GGT, but not liver stiffness (LS). For chronic systemic inflammatory, only TNF-α and IL-6 levels decreased significantly in the probiotic group. However, for lipid profiles, only TC was significantly reduced after probiotic treatment comparing mean changes from baseline. This study also mentioned that mono-probiotic strains did not prevent NAFLD. Therefore, the research confirmed the benefits of probiotics in improving liver enzymes and lipid profile in patients with NAFLD, whereas inflammation was not improved (85, 86). For obese patients with NAFLD, probiotics reduced ALT, AST, cholesterol, LDL-C, TG, and waist circumference but not BMI (79, 87). For adult NASH, probiotic mixture was showed to have no effect on BMI, lipid and glucose profile and atherogenic indexes (88).

The effect of probiotics on immune was assessed by Nor et al. They found that multi-strain probiotics (MCP^®^ BCMC^®^ strains), containing six different Lactobacillus and Bifidobacterium species, did not affect CD4+ or CD8 + T lymphocytes. The strains did neither influence steatosis, fibrosis, inflammation scores, ALT, TC, TG, and fasting glucose in NAFLD patients. The only positive result was that the strains stabilized the mucosal immune function in gut microenvironment and protected NAFLD patients against increased intestinal permeability (89). Only several studies were referred to the effect of probiotics on gut dysbiosis, which showed the gut microbiota and permeability tended toward a normal pattern after the treatment of probiotics (77, 80, 82, 89).

In the past 10 years, the clinical trials on probiotic treatment for NAFLD have been improved, however, the effects and beneficial roles are still in dispute. In the trials above, it is not difficult to find the contradictions, which may be interpreted by different study designs, the dosage of probiotic, types and duration of supplements, subjects under investigation and so on. In summary, probiotics may be beneficial to reduce transferase and partly lipid profiles only in the early stage of NAFLD. Long-term studies can provide a higher level of evidence, and whether probiotics can prevent NAFLD progression to liver fibrosis remain to be further explored.

## 4. Challenges and prospect

Recently, probiotics treatment has been a hot topic for metabolic diseases. Although abundant animal experiments have confirmed the beneficial effects of probiotics on NAFLD and the partial mechanism in this process, the clinical application is still conducted by evidence from clinical trials. Currently, there are huge challenges of probiotics treatment for NAFLD: (1) The selection of probiotics is difficult due to the diversity of probiotics strains. The differences of effects may be contributed by different strains. Current studies showed that muti-strains were more effective single strain. (2) Patients with early-stage NAFLD have been improved with decreased transferase after probiotics management. However, whether probiotics prevent the progression to liver fibrosis is unclear. (3) The duration of study and dosage of probiotics both influence the results of efficacy. Nevertheless, probiotics treatment is considered as the promising therapeutic strategy and hoped to be the effective, substantial, and common managements for NAFLD patients.

## Author contributions

CC wrote the manuscript. MS and XW proofread the manuscript. YY supervised the work and provided financial support. RZ designed the work and provided financial support. All authors contributed to the article and approved the submitted version.

## Funding

This work was supported by the National Natural Science Foundation of China (grant numbers 81974087, 81974086, and 82170701).

## Conflict of interest

The authors declare that the research was conducted in the absence of any commercial or financial relationships that could be construed as a potential conflict of interest.

## Publisher’s note

All claims expressed in this article are solely those of the authors and do not necessarily represent those of their affiliated organizations, or those of the publisher, the editors and the reviewers. Any product that may be evaluated in this article, or claim that may be made by its manufacturer, is not guaranteed or endorsed by the publisher.
